# Multiple Brain Abscesses Caused by Nocardia Beijingensis in a Patient With HIV Infection

**DOI:** 10.7759/cureus.25754

**Published:** 2022-06-08

**Authors:** Dehydra M Leon-Tavares, Graciela Hernández-Silva, Paulette Diaz-Lomeli, Areli Martínez-Gamboa, Bruno A Lopez-Luis

**Affiliations:** 1 Infectious Diseases, Hospital Centro Médico Nacional (CMN) La Raza, Mexico City, MEX; 2 Infectious Diseases, Hospital General Dr. Manuel Gea González, Mexico City, MEX; 3 Medical Microbiology, Laboratory of Microbiology, Instituto Nacional de Ciencias Médicas y Nutrición Salvador Zubirán, Mexico City, MEX; 4 Infectious Diseases, Instituto Nacional de Ciencias Médicas y Nutrición Salvador Zubirán, Mexico City, MEX

**Keywords:** nocardiosis, immunocompromised, hiv, cerebral abscess, nocardia

## Abstract

The etiologic agents of central nervous system infections in HIV-infected patients comprise a broad range of opportunistic pathogens. We presented a 49-year-old male patient with HIV infection and low adherence to antiretroviral therapy. He presented with multiple cerebral abscesses, and his microbiological diagnosis approach resulted in the isolation of *Nocardia beijingensis,* a species rarely reported in America. Central nervous system nocardial infection in HIV-infected patients should be considered, and a diagnosis at species level is mandatory because the antibiotic susceptibility profile varies among species.

## Introduction

Patients with HIV infection without antiretroviral treatment with low cluster of differentiation 4 (CD4) cell counts have a high risk of central nervous system infection for opportunistic pathogens such as *Toxoplasma gondii*, *Mycobacterium tuberculosis,* and *Cryptococcus neoformans*. However, *Nocardia* spp. are opportunistic pathogens that cause disseminated disease in immunocompromised patients and there are several reports in patients with HIV infection worldwide [1,2]. *Nocardia beijingensis* was initially reported in Asia, and the case we presented here corresponds to the first case reported in Mexico. We described in this report its microbiological features and the outcomes of a patient with extensive cerebral diseases caused by *Nocardia beijingensis.*

## Case presentation

A 49-year-old man, originally from Mexico City, was diagnosed with HIV in 2006. He has a history of abandoning antiretroviral treatment two years prior, with the resumption of therapy six months before the current condition, with efavirenz/emtricitabine/tenofovir and trimethoprim-sulfamethoxazole (TMP/SMX) with poor adherence. He had a history of post-traumatic left amaurosis. In 2018, he was admitted to the emergency room due to severe headache, gait instability, nausea, and vomiting. He was admitted with a fever of 38.3°C and the rest of his vital signs were normal. He was alert with unaltered cognitive functions; his pupils showed anisocoria, veering gait, neck stiffness, and the rest of the physical examination was without significant findings. Since people with HIV present with subacute neurological deficits, the differential diagnosis includes inflammatory, infectious, or neoplastic causes [3].

**Figure 1:**
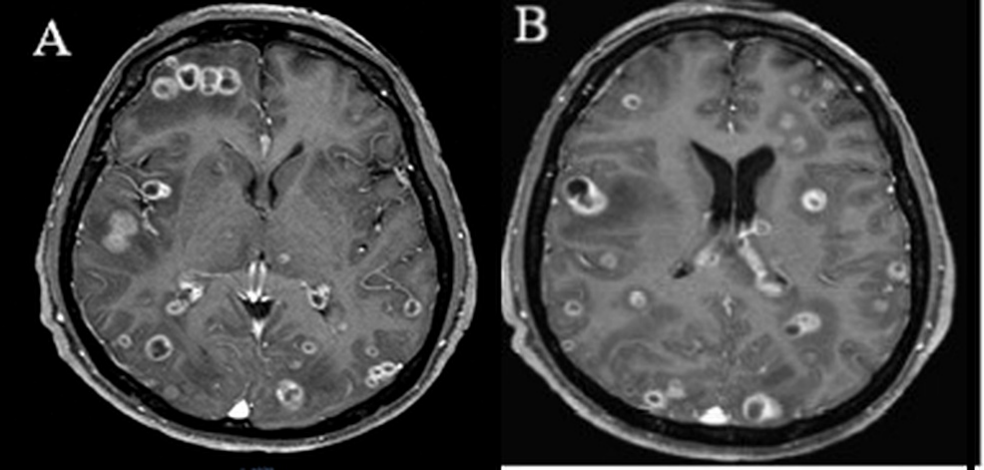
Axial T1-weighted magnetic resonance imaging (MRI) with contrast. (A) Multiple parenchymal lesions with ring enhancement in the right frontal, occipital lobes, and mesencephalon, with vasogenic edema; and (B) superior transversal image showing multiple ring-enhancing lesions, the parietal and occipital lobes are the most affected.

The laboratory findings showed the metabolic profile and the complete blood cell count results were unremarkable. The CD4 cell count was 66 cells/mm^3^ with an HIV viral load of 5806 copies/mL. The patient's chest x-ray was normal; therefore, making it less likely a frequent opportunistic pathogen such as tuberculosis in HIV-infected patients. A cranial magnetic resonance image showed multiple lesions with intra- and extra-axial ring enhancement without intracranial hypertension (Figures 1A, 1B).

**Figure 2:**
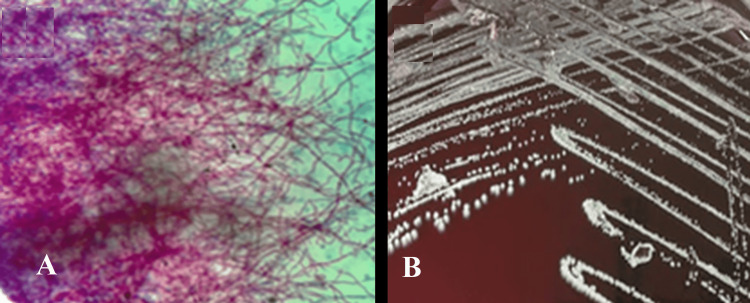
Microbiologic characteristics of Nocardia beijingensis. (A) Acid-fast modified Kinyoun stained smear showing thin branching filamentous acid-fast bacilli (x1000 magnification), and (B) small, whitish, rough colonies with scarce aerial hyphae growing in blood agar.

Since Toxoplasma encephalitis is the most common cause of intracranial lesion with ring enhancement, *Toxoplasma gondii* IgG antibodies and blood cultures for aerobic and anaerobic bacteria and fungi were required; the test results were negative [3]. Therefore, a lumbar puncture was performed, and the cerebrospinal fluid (CSF) showed pleocytosis at the expense of mononuclear cells (119 cells/mL with 65% mononuclear cells), slightly elevated protein, normal glucose, and negative Gram stain. India ink stain was negative, making less likely the diagnosis of neuromeningeal cryptococcosis. Also, acid-fast stain was negative; adenosine deaminase was 3 U/L, and polymerase chain reaction (PCR) for *Mycobacterium tuberculosis *was negative. A craniectomy with drainage of brain abscesses and biopsy were performed. The Gram stain of the biopsy showed branched Gram-positive bacilli and a positive Kinyoun modified acid-fast stain (Figure 2A). The sample was inoculated in blood and Sabouraud agar was incubated to 35-37°C showing growth on the ninth day (Figure 2B). Molecular tests identified the isolate; the 16S rRNA gene (500 nucleotides) and the secA1 gene (480 nucleotides) were amplified and sequenced. Sequence alignments in BLAST (Basic Local Alignment Search Tool) and MycoBank were performed [4,5]. The isolate was identified as *Nocardia beijingensis* with 100% (16S rRNA) and 99.7% (secA1) of similarity to the reference strain CNM20090803. The sequences obtained were deposited in GenBank with accession numbers MZ853573 (16S rRNA gene) and MZ882454 (secA1 gene).

After two weeks of treatment with imipenem and TMP/SMX intravenously, a susceptibility test was available, reporting resistance to imipenem, quinolones, and tetracyclines, with susceptibility to linezolid, TMP/SMX, and amoxicillin/clavulanic acid; thus, imipenem was discontinued and we continued for an additional three weeks intravenously with linezolid and TMP/SMX. A follow-up computed tomography scan was performed, which showed a marked decrease in lesions and significant neurological improvement. He was discharged home with oral management for 12 months with amoxicillin with clavulanic acid and TMP/SMX, with clinical and tomographic follow-up with no evidence of relapse.

## Discussion

The genus Nocardia belongs to the group of aerobic actinomycetes, and they are Gram-positive, branching filamentous bacilli, and slightly acid-fast bacteria. Nocardia spp. are ubiquitous environmental saprophytes in soil, organic matter, and aquatic habitats, including waste-water systems. Human infection usually arises from direct inoculation of the skin or soft tissues or by inhalation. It is an opportunistic pathogen most commonly affecting immunocompromised patients, although approximately one-third are immunocompetent [1]. The patients at highest risk are those with hematopoietic stem cell transplantation, solid organ transplantation, HIV infection, malignancy, and chronic glucocorticoid therapy [1]. Overall, the incidence of nocardiosis among patients with acquired immune deficiency syndrome is relatively low (0.1-0.4%) compared with other opportunistic diseases. The patients with a low CD4 T-cell count (less than 100 cells/mm^3^) are considered at the highest risk, with increased morbidity and mortality rates. Conventional TMP/SMX prophylaxis may reduce the rate of nocardial infection in transplant recipients and HIV-infected patients; however, cases have been reported in patients receiving such treatment or associated with poor adherence, as was reported by our patient [2].

Central nervous system (CNS) nocardiosis has a low prevalence but high morbidity and mortality [1,6]. Anagnostou et al. presented a review of 84 cases of CNS nocardiosis reported in the literature from January 2000 to December 2011; the most typical signs and symptoms of CNS nocardiosis were variable and included focal neurologic abnormalities (51%), headache (45%), fever (40%), altered mental status (36%), seizures (28%), visual changes (21%), nausea and vomiting (21%) [6]. Clinical manifestations usually result from local effects of granulomas or abscesses in the brain, mainly in the supratentorial region and, less commonly, the spinal cord or meninges [7]. Depending on the abscess phase, the lesion can be seen in a CT scan or magnetic resonance imaging as a single or double ring enhancement [8]. Approximately 54% of cases reported of Nocardia* *brain abscess were solitary lesions, while 38% of cases had multiple lesions [9]. The MRI of our patient showed broad encephalic lesions with ring enhancement in the right frontal, occipital lobes, and mesencephalon.

Clinically relevant isolates of Nocardia should be identified beyond the genus level because each species has its clinical spectrum of disease, sensitivity, and resistance profile to different antibiotics [10]. Mass spectrometry (matrix-assisted laser desorption/ionization-time of flight {MALDI-TOF} MS) is considered a reliable methodology that allows the identification of Nocardia isolates, reporting in 95.9% identification at the species level. The definitive identification method, mainly for uncommon species, is based on sequencing genes such as 16S rRNA, secA1, hsp65, gyrA, and rpoB [11].

*Nocardia beijingensis* was first described in China in 2001, phenotypically characterized and 16S rRNA gene sequence reported and classified within the Nocardia abscessus complex [11,12]. There have been reports of *Nocardia beijingensis* infection in HIV-positive patients from the United States [13] and Thailand [7] including in immunocompetent patients from Japan and Colombia [8,9]. There are reports of central nervous system infections caused by *Nocardia beijingensis*, mainly in male patients between 45 and 71 years of age with various comorbidities such as HIV infection, kidney transplant, diabetes mellitus, and even immunocompetent patients [7-10,13-15]. In those reports, the CNS was the primary source in four cases [8,9,13,14]. In our patient, other foci of Nocardia infection were ruled out, as was the absence of respiratory symptoms and a normal chest x-ray as well as absence of cutaneous lesions, so that his presentation was limited to the CNS.

The selection of antibiotics for empirical combination therapy should be based on Nocardia species. An option as a primary scheme would be TMP/SMX plus imipenem, considering adding amikacin in cases with multi-organ involvement. As alternative schemes, the use of meropenem plus linezolid has been mentioned. Imipenem showed better activity with less minimum inhibitory concentration (MIC) in vitro [16]. Our susceptibility test reported resistance to imipenem that was consistent with the study reported in Taiwan of 151 unduplicated Nocardia isolates; of which, nine were *Nocardia beijigensis* isolates, five were susceptible, and four had intermediate sensitivity to imipenem with an MIC between 0.5 and 8 ug/mL [17]. Another case report of an HIV-infected Hispanic patient with CNS infection by *Nocardia beijigensis* showed resistance to imipenem [13]. The *Nocardia beijigensis* isolates reported so far are susceptible to TMP/SMX [10,13,17]. However, treatment failure has been observed when used alone, especially in disseminated and central nervous system nocardiosis. Empirical combination with TMP/SMX, linezolid, or aminoglycosides is a reasonable decision since linezolid has excellent activity against all Nocardia species reported to date and has a CNS penetration of 60-70% [8,13,16,17]. The optimal duration of treatment for Nocardia infections is unclear and depends on the patient's clinical status and immune system.

## Conclusions

For the diagnosis of CNS in HIV-infected patients, an aggressive etiologic agent should be sought since atypical pathogens such as Nocardia* *spp. might be the opportunistic agent causing disease. In addition, appropriate speciation of Nocardia* *isolates and antimicrobial susceptibilities test allows a survey of new species and an optimal antimicrobial therapy.
